# The Role of Surface Exposed Lysine in Conformational Stability and Functional Properties of Lipase from *Staphylococcus* Family

**DOI:** 10.3390/molecules25173858

**Published:** 2020-08-25

**Authors:** Nurul Nadirah Ahmad, Nor Hafizah Ahmad Kamarudin, Adam Thean Chor Leow, Raja Noor Zaliha Raja Abd. Rahman

**Affiliations:** 1 Enzyme and Microbial Technology Research Center, Faculty of Biotechnology and Biomolecular Sciences, Universiti Putra Malaysia, Serdang Selangor 43400, Malaysia; nurulnadirahad@gmail.com (N.N.A.); adamleow@upm.edu.my (A.T.C.L.); rnzaliha@upm.edu.my (R.N.Z.R.A.R.); 2 Department of Microbiology, Faculty of Biotechnology and Biomolecular Sciences, Universiti Putra Malaysia, Serdang Selangor 43400, Malaysia; 3 Centre of Foundation Studies for Agricultural Science, Universiti Putra Malaysia, Serdang Selangor 43400, Malaysia; 4 Department of Cell and Molecular Biology, Faculty of Biotechnology and Biomolecular Sciences, Universiti Putra Malaysia, Serdang Selangor 43400, Malaysia

**Keywords:** charged residue, lysine, staphylococcal lipase, mutagenesis, enzyme stability

## Abstract

Surface charge residues have been recognized as one of the stability determinants in protein. In this study, we sought to compare and analyse the stability and conformational dynamics of staphylococcal lipase mutants with surface lysine mutation using computational and experimental methods. Three highly mutable and exposed lysine residues (Lys91, Lys177, Lys325) were targeted to generate six mutant lipases *in silico*. The model structures were simulated in water environment at 25 °C. Our simulations showed that the stability was compromised when Lys177 was substituted while mutation at position 91 and 325 improved the stability. To illustrate the putative alterations of enzyme stability in the stabilising mutants, we characterized single mutant K325G and double mutant K91A/K325G. Both mutants showed a 5 °C change in optimal temperature compared to their wild type. Single mutant K325G rendered a longer half-life at 25 °C (T_1/2_ = 21 h) while double mutant K91A/K325G retained only 40% of relative activity after 12 h incubation. The optimal pH for mutant K325G was shifted from 8 to 9 and similar substrate preference was observed for the wild type and two mutants. Our findings indicate that surface lysine mutation alters the enzymatic behaviour and, thus, rationalizes the functional effects of surface exposed lysine in conformational stability and activity of this lipase.

## 1. Introduction

Proteins are made up of different amino acids that carry unique properties and responsible for folding, function, stability, and solubility. Protein stability is a crucial property as it determines protein functionality. It is well accepted that factors, such as hydrogen bonds, hydrophobic effect, ion pairs, and salt bridges are essential to promote protein stability. Apart from the intramolecular interaction, surface charge has been considerably recognized as one of the important aspects that can alter protein properties [1,2].

In a typical globular protein, one third of the amino acids on the protein-water interface are charged residues [3]. Five amino acid residues (Glutamate, Aspartate, Arginine, Lysine, and Histidine) are typically known as charged residues and they are often found in an ionized state close to neutral pH. These residues are claimed to contribute to protein stability by electrostatic interaction or surface charge-charge interaction [4,5].

Lysine and arginine are positively charged basic residues that are frequently observed on protein surfaces. Both lysine and arginine exhibit high pK_a_ values; thus, allowing them to form stable ionic and electrostatic interaction. Knowledge on the pK_a_ values appears to be a promising strategy to improve thermostability by optimizing the charge–charge interactions [6]. The geometric structure of arginine residue, which allows interactions in three possible directions, has been reported to provide more stability to a protein structure than lysine [7]. Moreover, in (cation–pi) interactions with aromatic groups, a preference of Arg over Lys has been described [8,9]. Pezzullo and co-workers [10] suggested few charged residues that involved as surface ion pairs contributed to the thermal stability of *Alicyclobacillus acidocaldarius* esterase 2 (EST2).

In addition, charged residues appear to improve protein resilience towards aggregation at elevated temperature due to their interaction with surrounding solvents [11]. Higher lysine (and lower arginine) content has been suggested to correlate with protein solubility [12]. A study conducted by Sokaligam et al. [7] suggested that surface exposed lysine and arginine residues were significantly involved in folding and solubility of green fluorescent protein (GFP) protein and this is also true for many other cases [13,14]. In contrast, Højgaard and co-workers recently suggested that surface charged residues were not an absolute prerequisite in both in vivo folding and solubility [15].

Interestingly, charged residues that are found on the protein interaction interfaces are often conserved across the homologous family [16]. Lipases (E.C.3.1.1.3), in particular those from *Staphylococcus* sp. are uniquely composed of high number of charged residues especially lysine. Out of 20 amino acids, 20–25% are charged residues (positively and negatively charge) and lysine constitutes approximately 6–8% of the total amino acids found in the sequence. Meanwhile, a lower occurrence of lysine is observed in *Pseudomonas* and *Bacillus* lipases with 2–3% and 5–6%, respectively. To the best of our knowledge, given the case of staphylococcal lipases, the function of lysine has not been discussed.

Previously, a gene encoding for cold-adapted, organic solvent stable lipase from *Staphylococcus epidermidis* strain AT2 was isolated, cloned and expressed in an *Escherichia coli* system [17]. Interest in staphylococcal lipase has been ongoing since decades ago due to their ability to catalyse esterification, interesterification and transesterification in non-aqueous medium. Xie et al. [18] stated that several staphylococcal lipases have been successfully applied in esters production industry. Lipases from *S. capitis* was utilized in hair treatment formulation, whereas *Staphylococcus xylosus* lipase was used in production of fermented food such as ham and sausages. Meanwhile, Horchani et al. [19] also reported the potential of staphylococcal lipase in detergent industry owing to its highly alkaline and thermostable properties.

Because of their potential in diverse application, it is imperative to investigate the stability determinants to allow further manipulation. Moreover, the fact that staphylococcal lipase has a high number of lysine, it is presumed that this residue may play an important role in the stability and functionality. In this study, we sought to examine the effect of surface exposed residue mutation, particularly lysine to the stability of a mutant originated from a previous study [20]. This recombinant truncated lipase (herein designated as wild type, WT) is a monomeric protein of 386 residues with 22% of charged residues (lysine, arginine, aspartate, and glutamate) and lysine has the highest occurrence. *In silico* mutation was performed and analysed by molecular dynamics (MD) simulation. The effects of mutation on the activity and stability of selected mutant lipases were investigated by biochemical and biophysical approaches.

## 2. Results

### 2.1. Prediction and Identification of Mutation Sites

Lysine residues are positively charged basic amino acids and often found on protein surfaces. They take part in protein stability by several interactions, including the hydrogen bond formations, ionic interactions, and interaction with water molecules [4]. Multiple sequence alignment of WT lipase with several lipase sequences in the *Staphylococcus* family (Family I.6) was performed to assess the conservation pattern and prevalence of lysine in the family. Based on the alignment, most of the lysine residues are found conserved and only four residues (Lys91, Lys193, Lys372, and Lys380) are not conserved across the family. The staphylococcal lipase sequences were also aligned with other homologous sequences from the genus *Bacillus* and *Pseudomonas* and to our expectation, distribution of lysine in *Bacillus* and *Pseudomonas* lipases is random and not conserved (Figure S1).

To further understand the properties of this amino acid, each lysine residue was evaluated in terms of its position on secondary structure, solvent accessible surface area (SASA) value, and whether it was buried or surface exposed. Table 1 shows the distribution of 27 lysine residues, which corresponds to 7% out of the total amino acid composition. Lysine, threonine and leucine appear as the second most abundant residues, while glycine is the most abundant amino acid in the protein sequence with 11%. The occurrence of high glycine is positively correlated with the cold-adapted property of this enzyme. Glycine has been suggested to largely contribute to its conformational flexibility and high catalytic activity at low temperature [20].

Out of 27 lysines, four residues are found buried with SASA values below 50 Å, while others are surface exposed to a maximum SASA value of 183.9 Å^2^ (Table 1). The site of mutation was identified with the aid of HotSpot Wizard 2.0 (https://loschmidt.chemi.muni.cz/hotspotwizard/) (excluding the catalytic triad; Ser116, Asp307, His349 and all buried residues). This tool provides an automated identification of hot spots (high mutability) and design of smart libraries for protein stability engineering. Based on the prediction, K91, K177, and K325 were ranked as the top three lysine residues with high mutability score of 6, 9, and 9, respectively. The accessible surface area values obtained from HotSpot Wizard 2.0 showed that the three residues are surface exposed with SASA values ranged from 45.9 Å^2^ to 126.45 Å^2^. These three lysine residues, which are located at coil, helix and beta secondary structures, respectively were substituted to a neutral amino acid, alanine or glycine to generate three single mutants (K91A, K177A, K325G) and a random combination of the single mutations to produce three double mutants (K91A/K177A, K91A/K325G, and K177A/K325G) for comparative study. The selection of neutral residues, alanine or glycine were based on suggestion and frequencies of amino acid provided by HotSpot Wizard 2.0 tool.

### 2.2. Model Building and Validation of WT Lipase and Six Mutant Lipases

The amino acid sequence of WT lipase was aligned to the selected template (Protein Data Bank (PDB): 2HIH) with 51% identity (Figure S2). The secondary structure of target sequence was first generated to aid alignment correction and loop modelling, followed by building of side chains and optimization of rotamers, before subjected to steepest descent energy minimization using AMBER03 force field. The model of WT lipase was built. The same procedures were applied in building the predicted model of other mutant lipases.

**Figure 1:**
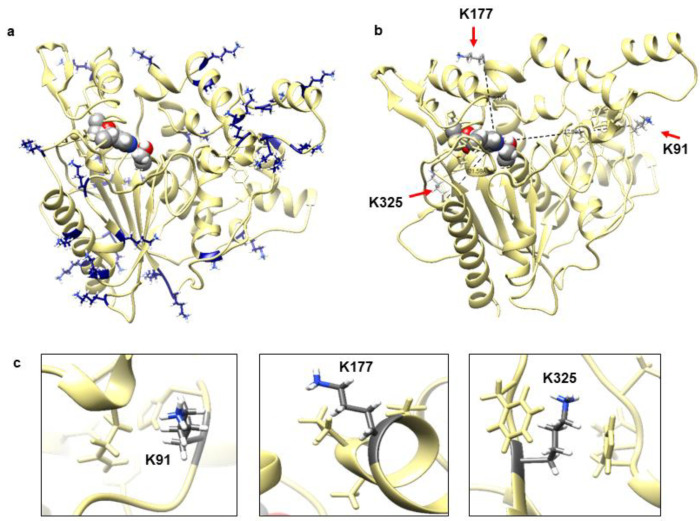
(**a**) Predicted model structure of WT lipase with catalytic triad in sphere shape located at the protein centre core. All lysine residues were distributed all over the protein surface were shown in heteroatoms colour. (**b**) The distance of three lysine residues (shown by red arrow) from catalytic triad (sphere shape heteroatom colour). (**c**) The location of three selected lysine (K91, K177, and K325) at coil, helix, and beta are shown in element colour with neighbouring residues. The figure was generated by Chimera v1.13.1.

Figure 1a shows that this lipase adopted an α/β hydrolase fold and a lid domain, which is commonly exhibited by lipase in superfamily of α/β hydrolases [21]. The overall three-dimensional (3D) structure is formed by 19 α-helices and nine β-strands; and conserved sequence motifs GHSMG include highly conserved Ser116, catalytic residue Asp307 and His349. Highly conserved GXSXG pentapeptide motif in lipase include a serine and histidine that hydrogen bonded to a glutamate or aspartate to form a catalytic triad and oxyanion hole [22,23]. The distribution of charged residues particularly lysine is also shown in Figure 1a. Most of the lysine residues were clearly seen exposed to the protein surface and some on the loop regions. Figure 1b,c show the specific location of the targeted residues. Lys91, Lys177, and Lys325 were located remote from the catalytic triad with a distance of 27.80 Å, 16.79 Å, and 21.59 Å, respectively.

The model structures of WT and six mutants were validated using PROCHECK (Ramachandran plot), ERRAT2, and QMEAN. The Ramachandran plot is one of the most extensively used structure validation tools to calculate each residue based on the phi-psi (φ/ψ) torsion angle exclude terminal residues [24]. Based on the Ramachandran Plot, (Table S1) high percentage of residues (>90%) was found in the most favoured region and additional allowed region for all models. None of the residues were present in disallowed region in except for mutant K91A/K177A but with a very low percentage, 0.3%. The results suggested that the models are of good quality.

In agreement with ERRAT2 analysis, the overall quality values for all models were more than 92% confirming the quality of the non-bonded interaction and indicated that the protein regions were correctly modelled. Finally, we used composite scoring function QMEAN to assess the global and local quality estimates of the models. This method is protein size independent to assess both entire oligomeric assemblies and isolated chain [25]. Five mutant lipases displayed a QMEAN Z-scores close to zero, in the range of −0.46 to −0.84, whereas double mutant K91A/K177A exhibited the lowest value with −1.68 (Table S2). A value higher than −4.0 indicates that the model structures is generally a comparable quality to the experimental structure of similar size thus can be used for subsequent analysis. In contrast, a model is considered low quality if the score is −4.0 or below. Analysis of C-β, all atoms, solvation and torsion also showed that all models displayed a value close to zero.

### 2.3. Superimposition and Secondary Structure Analysis

To examine the possibility of structural changes coupled to the surface lysine substitution, the predicted mutant structures were superimposed with the WT structure. The Root Mean Square Deviation (RMSD) values upon superimposition for six different mutants; K91A, K177A, K325G, and K91A/K177A were below 2.0 Å whereas for double mutants (K91A/K325G and K177A/K325G) the RMSD values were higher; 2.054 Å and 2.044 Å, respectively. The latter suggested that multiple substitution of charged to neutral residue resulted in a more apparent alteration to the overall structure.

### 2.4. Electrostatic Potential Maps

**Figure 2:**
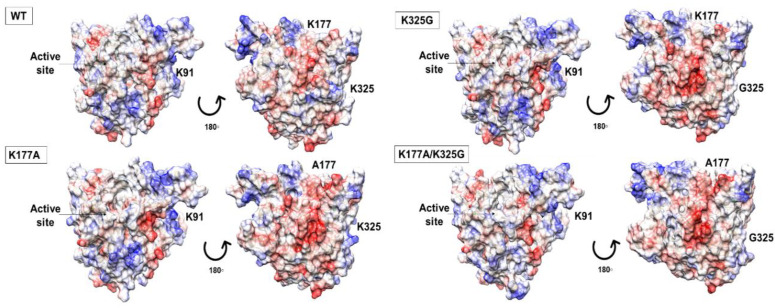
Electrostatic potential maps displayed on the surface of WT, mutant K177A, K325G, and K177A/K325G. The red colour indicated negatively charged region, positively charge region as blue and white colour defining neutral part of each lipase. The active site is circled and showed by arrow.

Electrostatic potential maps analysis showed that substitution of lysine to a neutral, non-polar residue did not profoundly alter the surface charge distribution of the mutants. The overall electrostatic potential maps are qualitatively similar, including the catalytic site. In the case of lipase, a negative potential in the active site seems to be associated with maximum activity towards triglycerides [26]. Similarly, our models showed a stretch of negative potential close to the catalytic residues. While WT and mutant K91A, K91A/K325G, and K91A/K177A shared a similar surface topology with almost an even distribution of positively and negatively charge (data not shown), the case is different for another three mutants. Mutant K177A, K325G, and K177/325G showed a more predominant negative charge surface compared to the WT (Figure 2).

### 2.5. In Silico Mutation and Molecular Dynamics (MD) Simulation in Water

**Figure 3:**
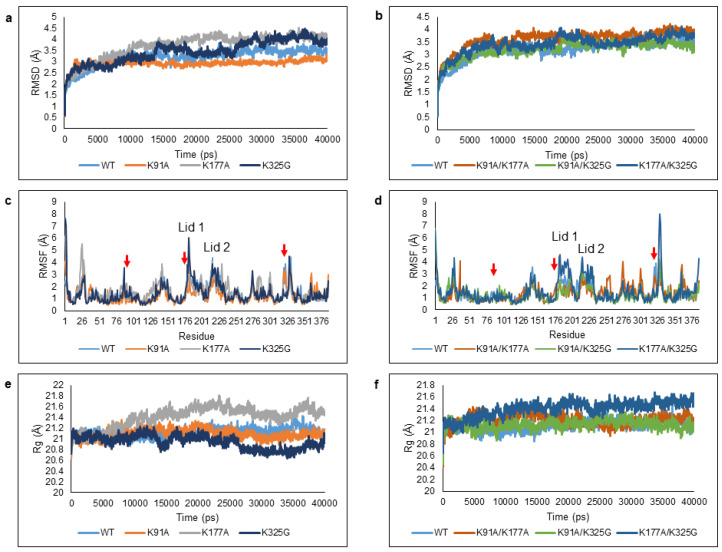
Deviations and fluctuations of the WT and mutant lipases when simulated in an aqueous environment. Trajectories of WT and mutant lipases were represented in different colour; (**a**,**b**) Root Mean Square Deviation (RMSD), (**c**,**d**) Root Mean Square Fluctuation (RMSF), and (**e**,**f**) Radius of gyration (Rg). The red arrows represent the position of K91, K177, and K325 in the RMSF graph.

To develop a mechanistic understanding on the effect of substitution via *in silico* mutation, we performed MD simulation in water for all mutants including the WT for comparative study. The simulations were carried out at 298 K (25 °C); the optimum temperature of WT over 40 ns trajectories and the outcomes were analysed by means of RMSD, Root Mean Square Fluctuation (RMSF), and Radius of gyration (Rg) (Figure 3).

RMSD calculations of protein backbone atoms were used to evaluate the structural stability of the WT and mutants. The values illustrated the divergence of enzyme structure relative to the reference structure (first frame) [27,28]. Figure 3a,b shows the RMSD profiles of all six mutants in comparison to the WT. Out of six mutant lipases, two mutants; K91A and K91A/K325G displayed lower RMSD values compared to the WT during the total course of simulation. This reflects an improvement in conformational stability. K91A was found most stable with very minimal Cα backbone deviation throughout 40 ns. The RMSD values of mutant K325G increased to 3.6 Å up to 35 ns but showed a decreased trend towards the end of simulation. Among single mutants, K177A was the least stable as it displayed the highest RMSD values throughout the simulation.

Site specific mutation can be further understood by analysing the average RMSF of Cα backbone per residue to observe the amino acid fluctuations over the trajectory (Figure 3c,d). The local flexibility can be detected according to the average interatomic distances between the atoms. The RMSF peak profile shows that most of the regions displayed moderate to high local movement mainly due to the presence of 27–30% of coil and 12–18% of turn in the structures. In total, we identified seven regions with high mobility; the N-terminal (Ala1-Val14), residues that stretched from Gly16-Val40, Glu126-Thr161, Thr181-Lys198 (Lid 1), Ser218-Ser229 (Lid 2), Ala268-Asn306, and His317-Trp338. Mutations at residue 91, 177, and 325 generally contributed to increased local flexibility at different regions of the structure. Double mutant K91A/K325G and K91A/K177A portrayed the lowest RMSF peak at the lid region, both Lid 1 and Lid 2. The terminal end especially N-terminal coil was highly flexible and this could also be observed in all mutants.

To examine the effect of surface mutation of the structural compactness, Rg was used to estimate the overall changes in total compactness and shape of the protein structure (Figure 3e,f). The outcomes allowed the overall dimension of enzymes to be understood and compared the compaction level of each enzyme [29]. Analysis of Rg values indicate that the conformation of the lipases was stable throughout 40 ns simulation where the overall structure and residue spatial arrangement remained almost constant (data not shown). The Rg value of K325G was the lowest among the mutants and WT. In contrast, mutant K177A and K177A/K325G became less rigid.

### 2.6. Intramolecular Interactions

**Figure 4:**
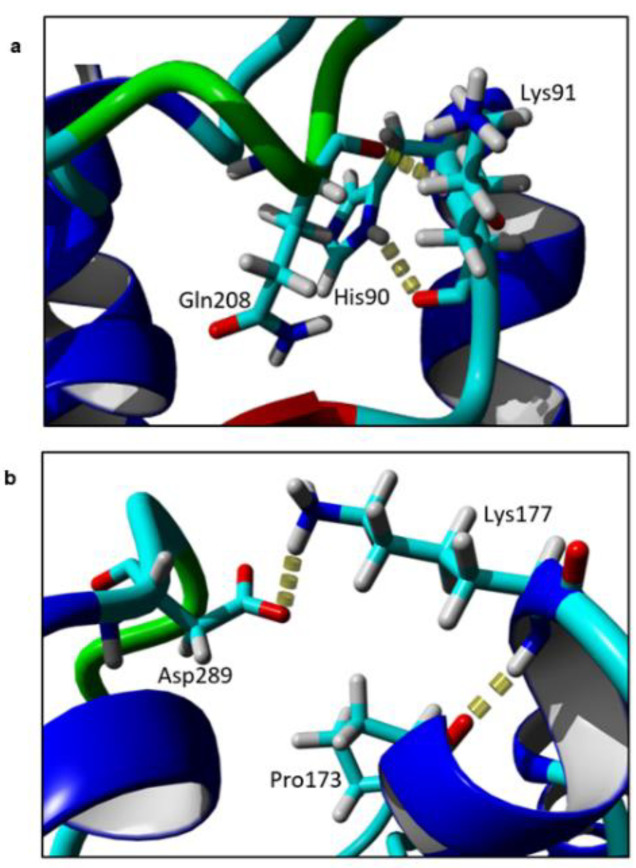
Hydrogen bond of (**a**) Lys91; and (**b**) Lys177 with neighbouring residues in element colour. The figure was generated by Yet Another Scientific Artificial Reality Application (YASARA).

Based on the models, the intramolecular interactions between the target residues and its neighbouring residues were identified. Two residues, Lys91 and Lys177, form contacts with their neighbouring residues by hydrogen bonds (Figure 4). Lys91 forms hydrogen bonds with two residues, His90 and Gln208. When substituted to alanine, no hydrogen bonds were observed in the mutants at the same region. Similarly, when Lys177 was replaced with Ala, the local hydrogen bonds between Lys177-Pro173 and Lys177-Asp289 were disrupted.

Apart from hydrogen bonds, electrostatic effects such as salt bridges play important role in protein stability. The interactions are highly varied, as they can be favourable or unfavourable [30]. This is due to the fact that the interaction can be attractive or repulsive and it requires ordering of the protein structure and desolvation of residue in water for them to interact to each other. A salt bridge or ion pair is formed based on the interaction of oppositely charged residues involving Asp, Glu, His, Lys and Arg. To examine the effect of mutation to salt bridge formation, analysis of salt bridge (or ion pairs) in each mutant was performed using ESBRI (http://bioinformatica.isa.cnr.it/ESBRI/introduction.html). According to ESBRI, only Lys177 forms salt bridges with Asp298 and Asp299 and no salt bridges are formed at Lys91 and Lys325 as seen in the WT.

### 2.7. Expression and Purification of Mutants

**Figure 5:**
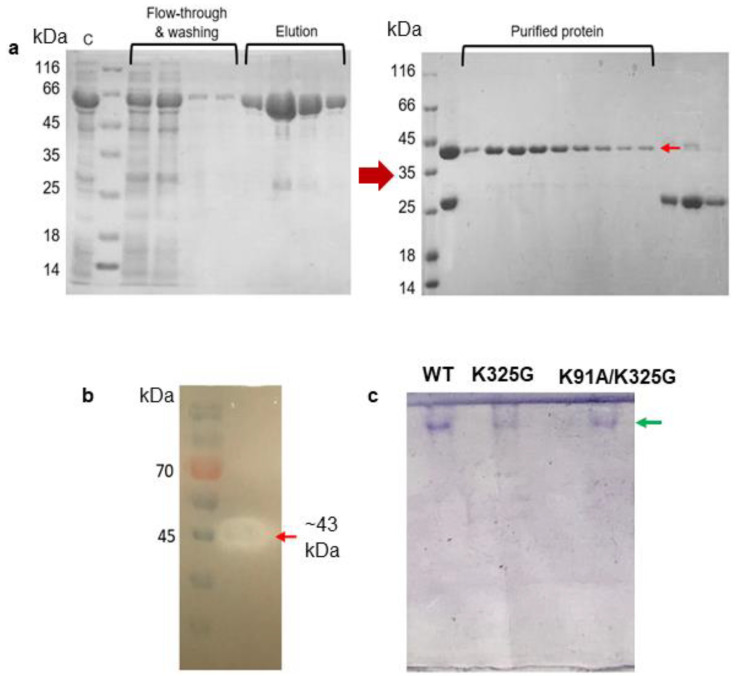
(**a**) SDS-PAGE (12%) analysis of two-steps purification of WT; (**b**) activity staining; and (**c**) native PAGE of purified WT, K325G and K91A/K325G. Symbols denote (C) crude enzyme, flow-through of impurities and washing step fractions. The elution fractions represented GST-tagged WT lipase (~69 KDa). GST tag was cleaved by PreScission Protease. Purified protein denotes purified WT (~43 KDa) shown by red arrow. GST tag was collected into later fractions (~26 kDa). A standard protein marker (Fisher Thermo Scientific, USA) was utilised. Activity staining with clearing zone on each purified mutant lipase (red arrows). Pre-stained protein ladder (Thermo Scientific, Waltham, MA, USA) was used as the marker. Native PAGE analysis showed a single protein band observed on the gel (shown by green arrow).

Following the simulation results, we selected two stabilising mutants for biochemical characterisation, one from the single mutants and another from the double mutants. The basis of selection was made by looking at mutants with improved stability from the MD simulation analysis. Mutant K325G and K91A/K325G were generated using overlap PCR method and confirmed by DNA sequencing (data not shown). Both mutants were expressed as intracellular recombinant Glutathione S-transferase (GST)-fused lipases (~69 kDa) and further purified using a two-step GST-affinity chromatography. The purification fold and yield of the WT and mutants were in the range of 4 to 5 and approximately 60%, respectively. A pure single band at the size of approximately 43 kDa was detected on SDS-PAGE gel after the second GST-affinity (Figure 5a). Reduction in size was due to cleavage of the GST tag by PreScission Protease. Purification of K325G and K91A/K325G yielded the similar SDS-PAGE profile (Figure S3). All tested lipases were further subjected to activity staining and Native PAGE. A clear halo zone, which matched the size of protein on SDS-PAGE gel, was observed on the tributyrin agar confirming the presence of lipolytic activity (Figure 5b). Meanwhile, in Figure 5c, a distinct protein band was observed in the Native PAGE analysis of each mutant and WT signifying the homogeneity of the proteins.

### 2.8. Effect of Temperature on Lipase Activity, Half-Life Study, and Thermal Unfolding

**Figure 6:**
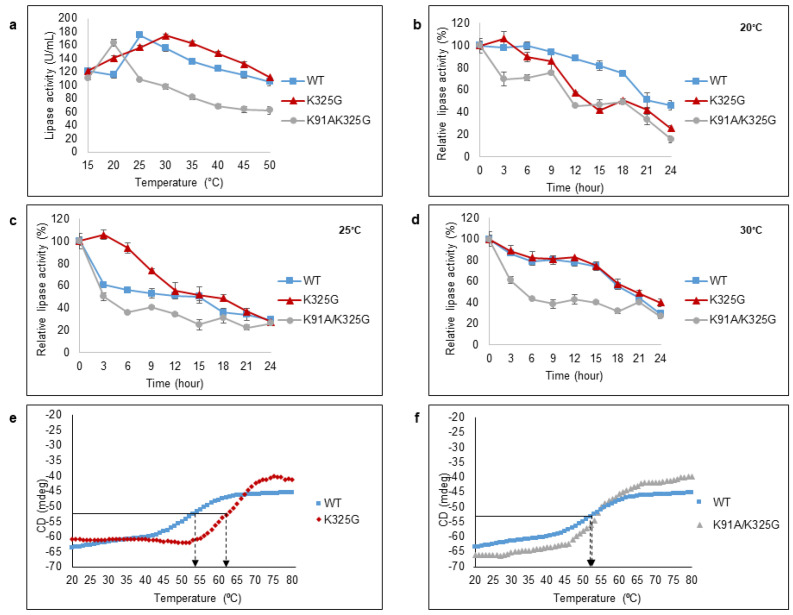
Effect of temperature on purified WT, K325G, and K91A/K325G lipase (**a**) activity and (**b**–**d**) half-life. Optimal temperature was determined by varying assay temperatures (15°C to 50°C). As for half-life, each lipase was incubated at different temperatures; 20 °C, 25 °C, and 30 °C for 24 h. The remaining activity was assayed at their optimal temperature. The protein concentration of each tested lipase was standardised to 0.5 mg/mL. Relative activity was calculated based on the control of the experiment. Non-incubated sample was regarded as the control (100%). Error bars denote the standard deviation of means (*n* = 3). Thermal unfolding (T_m_) curve of the WT with (**e**) K325G and (**f**) K91A/K325G. Heating rate was 1°C min^-1^. The arrows indicate the point of thermal unfolding of each enzyme. Symbols represent as follows; (■) WT, (♦) K325G, and (▲) K91A/K325G.

Considering temperature is one the main factors that influences protein functionality, the effect of mutations on both optimal temperature and stability was investigated. The lipolytic activity was measured using *p*-nitrophenyl palmitate as substrate. One unit of activity (U/mL) is defined as the rate of free *p*-nitrophenol release in 1 min under assay conditions. The thermal behaviour was measured at a temperature range of 15 °C to 50 °C (Figure 6a). Both mutants; K325G and K91A/K325G showed a 5 °C shift in optimal temperature compared to the WT. The maximum activity of K325G was 173.8 U/mL at 30 °C meanwhile double mutant K91A/K325G favoured a lower temperature, 20 °C. Mutant K91A/K325G displayed high sensitivity towards temperature above 25 °C with a drastic decline in lipolytic activity by 40%. Nevertheless, the three tested lipases retained their cold-adapted behaviour with high lipolytic activity at temperature below 30 °C. Cold-adapted lipases from other family such as LSK25 lipase from *Pseudomonas* sp. [31] and PT-11 lipase of *Oceanobacillus rekensis* [32] exhibited maximum activity at 30 °C and the activity dropped at temperature above 45 °C.

Half-life study was conducted at three different temperatures (20 °C, 25 °C and 30 °C) for 24 h (Figure 6b–d). Each temperature represents the optimum temperature of each tested lipase. At 20 °C, the WT retained more than 50% of the activity for 20 h whereas the half-life (t_1/2_) recorded for mutant K325G and K91A/K325G were within 12–15 h. At 25 °C and 30 °C, mutant K325G showed almost a similar trend of half-life to the WT but retained the activity more than 50%, 3 h longer than WT at 25 °C. On the contrary, mutant K91A/K325G displayed the shortest half-life at both temperatures. In fact, mutant K91A/K325G exhibited less than 40% of its relative activity after 12 h of exposure at every tested temperature. In general, with two positively charged residues removed, the effect is seen to be more detrimental than single mutation in terms of temperature stability.

Thermal unfolding (T_m_) of the mutants and WT were conducted using Circular Dichroism (CD) spectra (Figure 6e,f). It was measured at 222 nm, the wavelength that exhibited a large CD signal of α-helical from 20 °C to 80 °C. Double mutant K91A/K325G possess nearly identical T_m_ to the WT. Meanwhile the T_m_ of K325G was 62.95 °C, approximately 7–8 °C higher than the WT. The high T_m_ observed in K325G is positively correlated with higher activity compared to the WT and K91A/K325G. The data suggests that mutant K325G, with unexpectedly high T_m_ value, shows more of mesophilic enzyme characteristic.

### 2.9. Effect of pH on Lipase Activity and Stability

**Figure 7:**
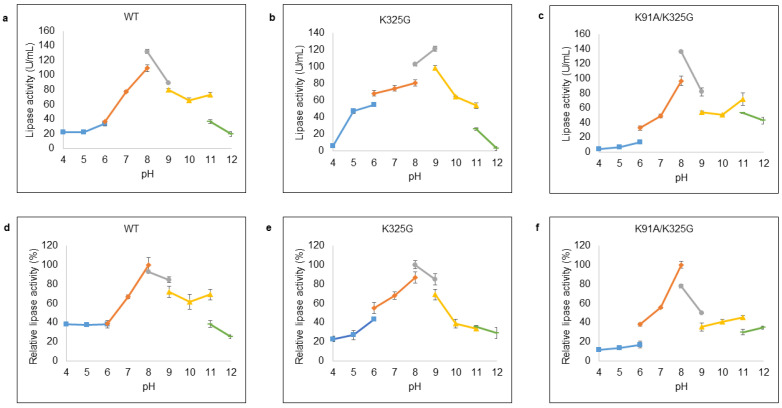
Effect of pH on lipase activity of purified (**a**) WT, (**b**) K325G, and (**c**) K91A/K325G. Optimum pH was identified by varying the pHs (4–12). Tested lipase was assayed at 25 °C for 10 min. The protein concentration of each tested lipase was standardised to 0.5 mg/mL. While, effect of pH on lipase stability of purified (**d**) WT, (**e**) K325G, and (**f**) K91A/K325G. The buffer systems used are as follows: sodium acetate (pH 4–6); sodium phosphate (pH 6–8); Tris-HCl (pH 8–9); glycine-OH (pH 9–11) and sodium hydrogen phosphate (pH 11–12). The purified lipase was pre-incubated at the tested pHs for 30 min. Relative activity was calculated based on the control of the experiment. Non-incubated sample as control (100%) were employed to calculate the relative activity. Error bars represent standard deviations of means (*n = 3*).

pH plays an important role in regulating the active site of enzyme. Optimal pH was examined at pH ranges from 4 to 12 with appropriate buffer systems. Figure 7a–c demonstrated that WT and K91A/K325G exhibited optimal activity at pH 8 with 132.01 U/mL and 136.05 U/mL, respectively, in Tris-HCl buffer. Meanwhile, K325G displayed maximum activity (121.91 U/mL) at pH 9 in the same buffer system. Severe deactivation was observed at extreme pH; both highly acidic (pH 4 to 5) and alkaline (pH 11 to 12).

In terms of stability, each lipase was found fairly stable at wide pH distribution as shown in Figure 7d–f. Mutant K325G was stable at pH ranging from pH 6 until pH 9 with maximum activity at pH 8 Tris-HCl buffer whereas K91A/K325G was stable at pH 7 to pH 8. Mutant K325G retained its lipolytic activity at broader pH profile compared to K91A/K325G and the lipolytic activity of both mutants diminished at highly acidic and alkaline pH.

### 2.10. Effects of Substrate on Lipase Activity

Enzymes are known to hydrolyse a wide range of substrates yet very selective and specific. Since the mutation sites are on the surface, we presumed that the effect of substitutions on substrate preference would be minor. In general, WT and mutants demonstrated highest lipolytic activity towards *p*-nitrophenyl myristate (C_14_) and a significant reduction of relative activity when incubated with short chain *p*-nitrophenyl esters (Table 2). Interestingly, both mutants showed excellent affinity towards long chain *p*-nitrophenyl esters. In relative to the WT mutant K325G showed enhancement of lipolytic activity in pNP decanoate by 140%, pNP dodecanoate (142%) and pNP myristate (128%). Mutant K91A/K325G, on the other hand, displayed 20–50% enhancement of lipolytic activity in C_10_–C_14_ substrates.

### 2.11. Effects of Organic Solvents on Lipase Stability

Purified WT and mutant lipases were incubated with 25% (*v/v*) organic solvents to assimilate the effects of organic solvents on lipolytic activities. Table 3 represents organic solvent stability profile of K325G and K91A/K325G in comparison to the WT, respectively. Generally, the mutant lipases demonstrated remarkable stability in polar compared to apolar organic solvents. Lipolytic activity of mutant K325G was observed to enhance in most polar solvents; DMSO, methanol, ethanol, and 1-propanol compared to the WT. Mutant K325G activity was observed in acetonitrile and chloroform with 100% increment. Meanwhile, the lipolytic activity of K325G in acetone was comparable to the WT. Almost all non-polar solvents showed a reduction in activity with 40–59% but greater activity than the WT. In comparison to the WT, mutant K91A/K325G was found to be more stable in ethanol and acetonitrile with increment of activity by 110% and 52%, respectively. Contrarily, in other organic solvents, mutant K91A/K325G denotes reduction of lipolytic activity. Treatment with non-polar solvents resulted in a slight increment of enzymatic activity of K91A/K325G as compared to the WT.

## 3. Discussion

The prevalence of lysine is a trend discernible in staphylococcal lipases but has not been clearly defined. The high occurrence of charged residues is often biased to thermostable proteins and known to impart stabilization by electrostatic or Van der Waals interaction. To unveil the role of lysine particularly those that are surface exposed, we used lipase from *Staphylococcus epidermidis* AT2 as a model. On the contrary, this enzyme is active at cold temperature. Lysine proportion is found similar among lipases of *Staphylococcus* family regardless of temperature preference. In this study, the three targeted surface-exposed lysines with high mutability score appeared at different secondary structures and two of the residues are conserved. The propensity of lysine to occupy the surface is well known due to its intrinsic polar hydrophilic property. Moreover, the fact that the ɛ-amino group of lysine can be involved in intramolecular interactions, surface charge mutation may likely cause alteration to structural stability and properties.

Through the simulations, RMSD was measured to observe the extent of conformational change between the initial structure and after 40 ns simulation. In all tested lipases, the average movement of backbone atoms is considered high with RMSD values more than 2Å. Mutant K177A, K325G, and K91A/K177A undergo relatively large motions whilst the remainders display stability improvement compared to the WT. The stability impairment in mutant K177A and K91A/K177A is presumed to be associated with disruption of stabilising interactions such as hydrogen bonds and salt bridges formed between the side chain of Lys91 and Lys177 with the neighbouring residues.

To explain further on structural stability correlations, a more detailed analysis was gleaned from RMSF spectra. A decrease in RMSF value of targeted residue was observed upon Lys-Ala/Gly mutation. Among the three positions, the largest amount of fluctuation occurred at regions close to where Lys177 and Lys325 resided. This is likely due to the position of the two residues near the flexible lid and a long coil close to the C-terminal, respectively. Residues that lie on the coil-beta-coil motif (residue 319–332) undergo high movement up to 4 Å and is presumed to partly contribute to the plasticity characteristic of this lipase. The surface lysine substitution is also seen to impart changes to the regions of close vicinity including the lid. It is generally known that the lid of lipases is one of the most flexible regions in the enzyme structure mainly due to its role in interfacial activation. Double mutations caused a more noticeable change in the lid movement. Mutant K177A/K325G showed high mobility around the lid especially at Lid 1 whereas for mutant K325G, K91A/K177A, and K91A/K325G, the lid is more rigid. In the case of cold-adapted enzymes, an increased flexibility has been suggested as the cold adaptation determinant, either localized close to the active site or a more distant part of the structure [33,34].

Consistent with RMSD and RMSF, mutant K177A and K177A/K325G displayed an increased in Rg value suggesting a decrease in structural compactness. A protein structure is considered to have a less tight packing when the Rg value is high and losing the structural compactness can lead to protein denaturation [35]. Protein rigidity is also associated with thermostability [36]. The correlation between thermostability and protein rigidity can affect the function of protein. Among all mutants, K325G is the most compact and rigid. Rigidity is important to sustain the integrity of native structure while some degree of flexibility is still required for lipase activity. However, in some studies, unlike K325G, the thermostable mutants from mesophilic *Bacillus* sp. native lipases demonstrated no changes in structural rigidity compared to its wild type lipases [37,38].

It is well accepted that a delicate balance between different weak intramolecular interactions such as salt-bridge formation, hydrogen bonding, cation-Pi and electrostatic interactions is essential for structural stability [34]. The correlation of hydrogen bond to structural stability has been widely reported [39,40] and the presence of more acid-basic surface ion pairs has been proposed to be one the stabilising factors seen in thermophilic orthologs [41]. Through our simulations, we deduce the same functional role of Lys177 in maintaining the conformational stability as it participates in both hydrogen bond and salt bridges. In agreement, introduction of additional salt bridges by seven mutations was reported to improve the stability of 1,4 α-glucan branching enzyme from *Geobacillus thermoglucosidans* STB02 [42]. Interestingly, Lys177 is highly conserved across the staphylococcal lipase family implicating an important role in staphylococcal lipase stability.

To confirm the simulation results, we characterised two stabilising mutants: K325G and K91A/K325G. Mutation of Lys325 led to an improved optimal temperature and longer half-life at 25 °C compared to the WT. This observation is in agreement with MD simulation results where the structure of K325G is more stable and rigid compared to the WT. Such improvement is possibly contributed by a significant reduction in local flexibility in the first coil of the coil-beta-coil motif where Lys325 is located. Replacement of lysine to alanine at the short three-residue beta strand stabilised the first coil comprising of residues 317–322 while the subsequent coil remained flexible. Yu et al. [43] reported that selection of flexible loops as mutation targets enhanced the thermostability of *E. coli* TK. This approach is also supported by other studies [44,45]. In addition, the position of two neighbouring lysine (Lys324 and Lys325) could be unfavourable due to repulsive force thus introduction of glycine might create a more favourable interaction between the adjacent residues.

As opposed to single residue substitution, double mutations resulted in a mutant that is more susceptible to the rise in temperature but catalytically active at lower temperature. Although single mutation at position 91 and 325 was initially predicted to enhance global or local stability; hence, thermal stability, a combined mutation pointed to the contrary when tested experimentally especially in functional temperature. Mutant K91A/K325G exhibited a 5 °C lower in optimal temperature and a shorter half-life at the three tested temperatures. The high sensitivity of double mutant at 30 °C could possibly due to interference of two hydrogen bonds at position 91. In addition, the fact that His90 is one of the residues that participates in coordinating Zn^2+^ in correct position, the loss of contacts between Lys91-His90 could indirectly affect the Zn^2+^ coordination. Zn^2+^ is known to play distinctive role in imparting structural stability [46,47].

For other biochemical properties tested, we observed only subtle changes in pH when compared with the WT. Similar to most staphylococcal lipases [18,48], these lipases favour Tris-HCl buffer system, having optimum activity at pH 8–9. The mutants generally retained the preference in alkaline condition for its optimum activity and stability. For substrate preference, mutations are less likely disruptive since they are positioned away from the catalytic triad. WT, mutant K325G, and K91A/K325G were able to hydrolyse a broad range of *p*-nitrophenyl esters, favouring the long-chain *p*-nitrophenyl esters (C_10_–C_16_). Classically, a true lipase is capable of hydrolysing long chain triglyceride (C > 10) [49], which is in agreement with our results. Staphylococcal lipases are known to exhibit a wide range of substrate preferences [18]. Similar to our results, *Staphylococcus aureus* strain ALA1 (SAL4) lipase efficiently hydrolyses long chain triacylglycerols [49]. On the contrary, *Staphylococcus aureus* lipase B56, the enzyme prefers to hydrolyse short and medium chain (C_2_–C_8_) triacylglycerols [50].

In term of solvent stability, the mutant lipases exhibited remarkable stability in polar compared to apolar organic solvents. Most enzymes are known to inactivate in polar solvent. This is due to their high degree of partitioning, disruptive of native hydrogen bonds, and strip off the essential water layer [51]. Therefore, it is notable that these mutants demonstrate an increase stability in polar solvents. Several studies reported only on solvent tolerant staphylococcal lipase in apolar solvents. *S. warneri* lipase shows increase stability in petroleum ether, *n*-hexane, cyclohexane, benzene and toluene while decrease in activity in most polar solvents [52]. However, similar to our results, 6B lipase from *Bacillus subtilis* exhibited an increase in activity in the presence of DMSO, methanol and isopropanol [53]. Sinha and Khare (2014) also reported that moderately halophilic *Bacillus* sp. EMB9 protease showed stability in various concentrations of polar organic solvents especially in the presence of 25% (*v/v*) of methanol and ethanol.

## 4. Materials and Methods

### 4.1. Template Selection

Template search was carried out by using Position Specific Iterated Blast (PSIBLAST) (http://www.ncbi.nlm.nih.gov/blast/) based on Protein Data Bank (PDB) database showing 51% identity similarity and 98% of query coverage to lipase crystal structure of *Staphylococcus hyicus* (PDB: 2HIH) [46] indicating the highest sequence identity to WT lipase. Hence, *S. hyicus* lipase was chosen as the template.

### 4.2. Structure Prediction and Evaluation of Protein Models

Comparative study and analysis of WT lipase and six mutants (single mutants: K91A, K177A, K325G; and double mutants: K91A/K177A, K91A/K325G, and K177A/K325G) were conducted via automated comparative modelling by a program called Yet Another Scientific Artificial Reality Application (YASARA). The WT lipase and template sequences were aligned prior to homology modelling using EMBL-EBI (The European Bioinformatics Institute) Clustal Omega (https://www.ebi.ac.uk/Tools/msa/clustalo/). Backbone, loop and side chain of protein models were built, optimized and fine-tuned to achieve the finest model quality. Simple minimization using AMBER03 force field was carried out and the models were refined in explicit solvent molecules. The final predicted model of WT and six mutants were evaluated by means of PROCHECK (Ramachandran Plot) (www.ebi.ac.uk/thornton-srv/software/PROCHECK), ERRAT2 (http://nihserver.mbi.ucla.edu/ERRATv2), and QMEAN (https://swissmodel.expasy.org/qmean/).

### 4.3. Secondary Structure Analysis

Analysis of secondary structure was done using a web server PDBSum (http://www.ebi.ac.uk/thornton-srv/databases/cgi-bin/pdbsum/GetPage.pl?pdbcode=index.html). The PDB files of all models were submitted to the web server to obtain the essential structural information.

### 4.4. Molecular Dynamics Simulations Setup

YASARA software version 17.4.17 was used to perform the simulations. A cubic simulation box (90° on every axis) was placed around the protein with 5.0 Å of distance around all atoms and the dimensions of the simulation box were determined automatically using the default parameter due to the size and amount of solvent molecules. The boundary conditions were set up to periodic in the x, y and z directions. Simulation was performed at pH 8; the optimum pH of the WT while the ion concentration (NaCl) was set to 0.9%. The number of water molecules was adjusted following the experimental density at 298.15 K equivalent to 25 °C. The pressure inside the simulation box rescaled until 0.997 g/cm^3^ of the experimental density was reached. Long-range electrostatics was treated using particle mesh Ewald method with a cut-off of 10.486 Å. The condition was kept constant for all variants and the WT enzyme. All simulations were carried out using the AMBER03 force field. A productive simulation of 40 ns for each of them was collected with a 1 fs time-step and conformations were stored every 50 ps [54].

### 4.5. Molecular Dynamics Simulations Analysis

The main chain Root Mean Square Deviation (RMSD) was calculated for each simulation using the starting structure as a reference in probing the structural stability during the course of simulation time. The profile of the main chain Root Mean Square Fluctuation (RMSF) per residue was averaged for each trajectory. Radius of gyration (Rg) was also calculated and analysed for comparative study of the mutants.

### 4.6. Construction of Mutants

Two mutants with improved stability were identified and constructed using the overlap PCR method. Three sets of primers (one set of native primers and one set of mutagenic primers of each mutant) were used. a) Native forward: 5′ CG GAA TTC GCA GCA ATG GCG CAA GCT CAA TAT 3′ and reverse: 5′ CCG CTC GAG TCA CTA ACC ATC TAG CTC TTC 3′; b) K91A forward: 5′ GCA AAA TAT GGT CAC GCG CGT TAT GGC AG 3′ and reverse: 5′ CT GCC ATA ACG CGC GTG ACC ATA TTT TGC 3′ (underlined is mutation point K91 to A); c) K325G forward: 5′ GAA GCA TTT AAG GGG GTA GGT ATG ATG AAC 3′ and reverse: 5′ GTT CAT CAT ACC TAC CCC CTT AAA TGC TTC 3′ (underlined is mutation point K325 to G). DNA fragments of the mutants were generated in the first round PCR and utilised in the second round PCR as the template to amplify the whole sequence consisting the mutation point. The mutated gene was amplified and transformed into expression vector, pGEX-6p-1, which carry glutathione S-transferase (GST) tag into *E. coli* DH5α and *E. coli* BL21 (DE3).

### 4.7. Expression and Purification of Mutant Lipases

Expression of WT, K325G and K91A/K325G were carried out following the method described in the previous work [20]. The mutants were expressed following the optimum condition (OD_600_ = 0.6, IPTG concentration = 0.5 mM and induction at 16 °C for 16 h). The bacterial culture was centrifuged at 4 °C, 10,000× *g* for 20 min. The pellet was kept at −20 °C.

Purification of the WT and mutants was conducted by two-step affinity chromatography using glutathione sepharose resin. Bacterial pellet was resuspended in pre-chilled binding buffer (50 mM PBS pH 7.4) for sonification (duty cycle: 30, output 2). The sample was cleared by centrifugation at 10,000× *g* for 30 min. The supernatant (soluble fraction) was collected and loaded into GST-affinity column and the target protein was eluted using elution buffer (50 mM Tris-HCl, 10 mM reduced glutathione pH 8). The fusion protein was then cleaved using PreScission Protease in cleavage buffer (50 mM Tris-HCl, 0.1 M NaCl pH 8) for 18 h. Second GST-affinity chromatography was conducted to collect the target protein from the flow through fractions and further dialysed against the storage buffer, 20 mM Tris-HCl pH 8. Further analysis was performed using SDS-PAGE, activity staining, and Native PAGE to confirm the purity of the protein.

### 4.8. Optimal Temperature and Half-Life

Changes in the optimum temperature of mutants were studied by assaying the purified lipases at different temperature ranging from 15 °C to 50 °C with 5 °C intervals for 10 min using *p*-nitrophenyl palmitate (C_16_) as the substrate. The final protein concentration was 0.5 mg/mL for all tested lipases. Absorbance reading at A_410_ was measured. Half-life study was determined by pre-incubating the purified lipase at respective temperatures (20 °C, 25 °C, 30 °C) for 24 h. Samples were removed at every 3 h interval and assayed at their optimum temperature. Sample at 0 min was regarded as the control (100%).

### 4.9. Optimum pH and pH Stability

The effects of various pH on the lipolytic activity and stability were measured in different buffer systems with pH ranging from pH 4 to 12. The buffer system used were sodium acetate (pH 4–6), sodium phosphate (pH 6–8), Tris-HCl (pH 8–9), Glycine/NaOH (pH 9–11), and sodium hydrogen phosphate (pH 11–12). Gum Arabic (0.1 (*w/v*)) was added into the mixture and subsequently assayed for 10 min at 25 °C. The final protein concentration was 0.5 mg/mL for all tested lipases. To examine the pH stability profile, purified lipase was pre-incubated in the tested buffer system for 30 min, and assayed at 25 °C for 10 min. Lipase activity was measured by measuring the absorbance at A_410_.

### 4.10. Substrate Determination

Various *p*-nitrophenyl substrates with different chain length ranging from C_2_ to C_16_ were used to determine the changes in substrate selectivity profile of the WT and two mutant lipases. The mixture was incubated for 10 min and assayed using standard *p*NP assay. The final protein concentration was 0.5 mg/mL for all tested lipases. *p*-nitrophenyl palmitate (C_16_) was regarded as the control (100%) to calculate the relative activity measured at A_410_.

### 4.11. Organic Solvent Stability

Organic solvent stability of WT, K325G, and K91A/K325G was studied by pre-incubating lipases in 25% (*v/v*) of various solvents with different log *P*. Incubation in DMSO (−1.3), methanol (−0.76), acetonitrile (−0.33) ethanol (−0.24), acetone (−0.24), 1-propanol (0.28), diethyl ether (0.85), chloroform (2.0), benzene (2.0), toluene (2.5), octanol (2.9), xylene (3.1), *n*-hexane (3.5), and *n*-heptane (4.0) was carried out for 30 min at 20 °C. Reaction mixture without solvent was regarded as control (100%). The mixtures were assayed for 10 min at 25 °C and residual activity of lipases were calculated.

### 4.12. Thermal Unfolding (T_m_)

Thermal unfolding was determined by using CD spectra (JASCO J-1500 CD spectropolarimeter). The spectrum was measured at 222 nm from 20 °C to 80 °C with 1 °C/min heating rate using PTC-510 Peltier thermostated cell holder. Protein was prepared at 1 mg/mL in 10 mM sodium phosphate buffer pH 7 and the measurement was repeated three times.

## 5. Conclusions

The structural and dynamics study of the WT and mutant lipases provide insights on the structural properties and behaviour of the protein conformation when simulated in water environment. Evidently, mutation on surface exposed lysine attributed to changes in the stability behaviour. Replacement of lysine to a smaller amino acid resulted in both stabilising and destabilising effects. Though our *in silico* mutation suggested that single and double mutants (K325G and K91/K325G) were stable, our experimental work implied that a combined mutation could accompany with stability trade-off even by combining the most stabilising single mutants. On another note, we proved that surface exposed lysine can be considered as one of the stability determinants in staphylococcal lipase.

## Figures and Tables

**Table 1:** Analysis of 27 lysine residues in the wild type (WT) lipase based on SASA value, secondary structure, buried/exposed and hydrogen bond. The values of SASA and the position of each residue whether buried/exposed were analysed by HotSpot Wizard 2.0. Meanwhile, the secondary structure and local hydrogen bond number were analysed using Chimera 1.13.1.

Position of Lysine	SASA (Å)	Secondary Structure	Buried/Exposed	Hydrogen Bond
**K6**	119.11	Coil	Exposed	3
**K37**	46.29	Coil	Buried	3
**K41**	77.02	Helix	Exposed	3
**K46**	136.85	Helix	Exposed	2
**K74**	63.22	Helix	Exposed	2
**K87**	129.01	Helix	Exposed	2
**K91**	112.3	Coil	Exposed	2
**K108**	109.73	Turn	Exposed	2
**K109**	27.37	Coil	Buried	0
**K152**	112.61	Coil	Exposed	2
**K177**	132.27	Helix	Exposed	2
**K182**	183.9	Helix	Exposed	1
**K185**	33.64	Helix	Buried	2
**K193**	41.61	Helix	Buried	3
**K198**	135.04	Helix	Exposed	1
**K212**	81.88	Coil	Exposed	1
**K214**	100.62	Coil	Exposed	1
**K224**	130.45	Helix	Exposed	2
**K230**	126.26	Coil	Exposed	2
**K249**	72.22	Helix	Exposed	1
**K301**	138.4	Coil	Exposed	1
**K324**	69.33	Beta	Exposed	3
**K325**	99.15	Beta	Exposed	0
**K335**	93.06	Coil	Exposed	1
**K361**	108.64	Coil	Exposed	1
**K372**	73.51	Helix	Exposed	2
**K380**	90.4	Helix	Exposed	3

**Table 2:** Effect of substrates with various chain length on purified WT, K325G, and K91A/K325G lipase activity.

p-NP	Relative Lipase Activity (%)		
	WT	K325G	K91A/K325G
C_2_	40.8 ± 1.9	34.5 ± 0.2	29.9 ± 3.8
C_4_	31.8 ± 0.5	48.1 ± 1.5	26.9 ± 0.5
C_10_	113.5 ± 3.0	253.8 ± 1.2	124.1 ± 2.2
C_12_	132.0 ± 2.5	274.1 ± 0.7	144.3 ± 2.1
C_14_	148.0 ± 1.2	276.2 ± 1.1	151.3 ± 5.7
C_16_	100.0 ± 3.4	100.0 ± 1.4	100.0 ± 0.8

Relative lipase activity was calculated based on the control of the experiment. *p*-nitrophenyl palmitate (C_16_) was regarded as the control (100%). The protein concentration of each tested lipase was standardised to 0.5 mg/mL.

**Table 3:** Effect of organic solvents on WT, K325G, and K91A/K325G lipase activity.

Organic Solvent (*Log P*)	Relative Activity (%)		
	WT	K325G	K91A/K325G
**Control**	100.0	100.0	100.0
**DMSO (−1.3)**	164.51 ± 1.4	244.7 ± 4.7	47.37 ± 2.2
**Methanol (−0.76)**	87.95 ± 5.5	160.89 ± 4.8	66.97 ± 3.0
**Acetonitrile (−0.33)**	25.76 ± 3.5	182.43 ± 3.5	78.44 ± 0.8
**Ethanol (−0.24)**	35.09 ± 4.8	117.66 ± 1.3	147.1 ± 8.1
**Acetone (−0.24)**	207.32 ± 5.9	204.61 ± 6.1	169.92 ± 7.6
**1-Propanol (0.28)**	12.31 ± 2.4	82.59 ± 1.8	39.06 ± 2.4
**Diethyl ether (0.85)**	89.16 ± 6.6	61.51 ± 2.5	59.71 ± 0.4
**Chloroform (2.0)**	37.96 ± 3.7	142.11 ± 2.1	38.02 ± 2.9
**Benzene (2.0)**	36.10 ± 1.3	59.45 ± 1.7	35.12 ± 2.1
**Toluene (2.5)**	22.61 ± 1.7	58.14 ± 1.9	50.15 ± 1.4
**Octanol (2.9)**	11.75 ± 1.8	46.29 ± 2.5	35.37 ± 1.9
**Xylene (3.1)**	22.97 ± 3.3	40.85 ± 0.7	54.35 ± 0.8
***n*** **-Hexane (3.5)**	31.13 ± 3.2	45.01 ± 1.5	32.71 ± 2.3
***n*** **-Heptane (4.0)**	30.74 ± 3.2	47.63 ± 0.7	30.05 ± 0.4

Purified enzymes were pre-treated with 25% (*v/v*) of tested organic solvents for 30 min followed by standard lipase assay at 25 °C for 10 min. Lipolytic activity in aqueous solution (without organic solvent) served as the control reaction (100%). Error bars represent standard deviation of means (*n* = 3).

## Supplementary Materials

The following are available online. Figure S1: Multiple sequence alignment of WT lipase to other Staphyloccocal lipase, *Bacillus* and *Pseudomonas*; Figure S2: Sequence alignment of WT lipase and template, 2HIH; Figure S3: SDS-PAGE (12%) analysis of two-steps purifications (a) K325G and (b) K91A/K325G; Table S1: Predicted structure validation of WT and mutant lipases based on Ramachandran plot and Errat2; Table S2: QMEAN analysis of WT and mutant lipases.
