# Supplementary material for: The Role of Surface Exposed Lysine in Conformational Stability and Functional Properties of Lipase from Staphylococcus Family
Source: Molecules. 2020 Aug 25;25(17):3858. doi: 10.3390/molecules25173858 (PMC7504586; doi:10.3390/molecules25173858)
Supplement: Supplementary file 1 [file molecules-25-03858-s001.pdf]

molecules

Article

The role of surface exposed lysine in conformational stability and functional properties of lipase from staphylococcus family

Nurul Nadirah Ahmad [1], Nor Hafizah Ahmad Kamarudin [1,2,*] Adam Thean Chor Leow [1,3], and Raja Noor Zaliha Raja Abd. Rahman [1,4]

1 Enzyme and Microbial Technology Research Center, Faculty of Biotechnology and Biomolecular Sciences, Universiti Putra Malaysia, Serdang Selangor 43400, Malaysia; nurulnadirahad@gmail.com (N. N. A); adamleow@upm.edu.my (T. C. L); rnzaliha@upm.edu.my (R. N. Z. R. A. R);

2 Centre of Foundation Studies for Agricultural Science, Universiti Putra Malaysia, Serdang Selangor 43400, Malaysia;

3 Department of Cell and Molecular Biology, Faculty of Biotechnology and Biomolecular Sciences, Universiti Putra Malaysia, Serdang Selangor 43400, Malaysia;

4 Department of Microbiology, Faculty of Biotechnology and Biomolecular Sciences, Universiti Putra Malaysia, Serdang Selangor 43400, Malaysia

* Correspondence: hafizah_kamar@upm.edu.my; Tel.: +60397696966; Fax: +60397696997

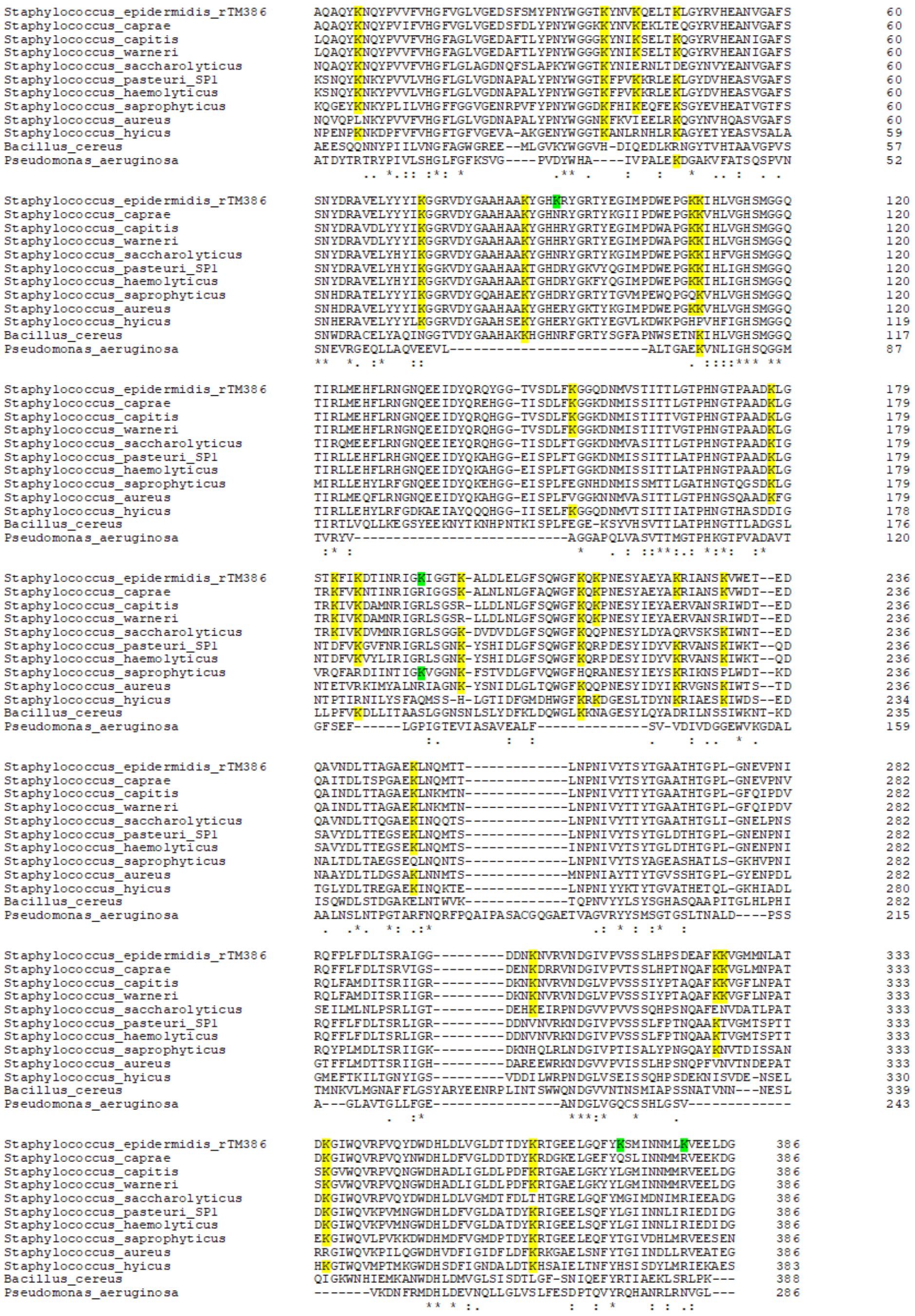

Figure S1. Multiple sequence alignment of WT lipase to other Staphylococcal lipase (Family I.6) and other genera of *Bacillus* and *Pseudomonas*. The highlighted in yellow colour showed lysine residue (highly conserved or non-conserved) across the various species of *Staphylococcus*; and *Bacillus* and *Pseudomonas* genera.

```
WT      --------------------------------------------AQAQYKNQYPVVFVHG  16
2HIH    MARIRARGSSRVDVPKENTTAQNKFTSQASDKKPTVKAAPEAVQNPENPKNKDPFVFVHG  60
                                                       : **: *.*****

WT      FVGLVGEDSFSMYPNYWGGTKYNVKQELTKLGYRVHEANVGAFSSNYDRAVELYYYIKGG  76
2HIH    FTGFVGEVA-AKGENYWGGTKANLRNHLRKAGYETYEASVSALASNHERAVELYYYLKGG  119
        *.*:*** : :   ******* *:::.* * **..:**.*.*::**::********:***

WT      RVDYGAAHAAKYGHKRYGRTYEGIMPDWEPGKKIHLVGHSMGGQTIRLMEHFLRNGNQEE  136
2HIH    RVDYGAAHSEKYGHERYGKTYEGVLKDWKPGHPVHFIGHSMGGQTIRLLEHYLRFGDKAE  179
        ********: ****:***:****:: **:**: :*::***********:**:** *:: *

WT      IDYQRQYGGTVSDLFKGGQDNMVSTITTLGTPHNGTPAADKLGSTKFIKDTINRIGKIGG  196
2HIH    IAYQQQHGGIISELFKGGQDNMVTSITTIATPHNGTHASDDIGNTPTIRNILYSFAQMSS  239
        * **:*:** :*:**********::***:.****** *:*.:*.*  *:: :  :.::..

WT      TKALDLELGFSQWGFKQKPNESYAEYAKRIANSKVWETEDQAVNDLTTAGAEKLNQMTTL  256
2HIH    -HLGTIDFGMDHWGFKRKDGESLTDYNKRIAESKIWDSEDTGLYDLTREGAEKINQKTEL  298
         :   :::*:.:****:* .** ::* ****:**:*::** .: ***  ****:** * *

WT      NPNIVYTSYTGAATHTGPLGNEVPNIRQFPLFDLTSRAIGGDDNKNVRVNDGIVPVSSSL  316
2HIH    NPNIYYKTYTGVATHETQLGKHIADLGMEFTKILTGNYIGSVDDILWRPNDGLVSEISSQ  358
        **** *.:***.***   **:.: ::       **.. **. *:   * ***:*   **

WT      HPSDEAFKKVGMMNLATDKGIWQVRPVQYDWDHLDLVGLDTTDYKRTGEELGQFYKSMIN  376
2HIH    HPSDEKNISVD-ENSELHKGTWQVMPTMKGWDHSDFIGNDALDTKHSAIELTNFYHSISD  417
        *****   .*.  *   .** *** *.  .*** *::* *: * *::. ** :**:*: :

WT      NMLKVEELDG----        386
2HIH    YLMRIEKAESTKNA        431
         ::::*: :.
```

Figure S2. Sequence alignment of WT lipase and the template, *S. hyicus* lipase (PBD ID: 2HIH). The conserved pentapeptide region (GXSXG) is shown in the red box. Symbols are indicated as follows: (*) fully conserved residue; (:) conservation of strong groups; and (.) conservation of weak group.

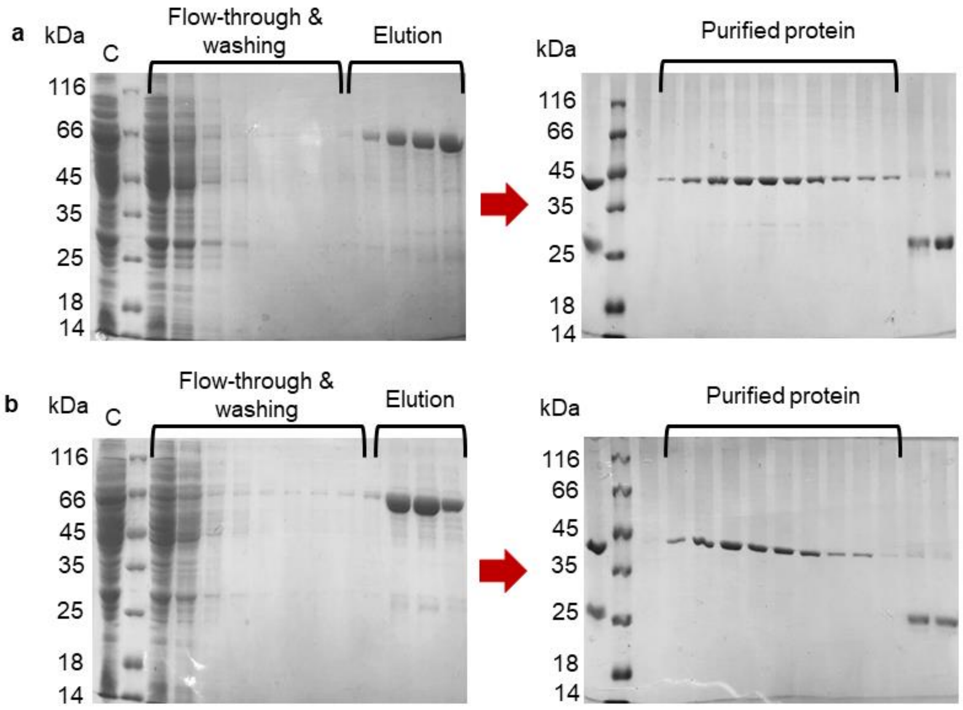

Figure S3. SDS-PAGE (12%) analysis of two-steps purification of **(a)** K325G and **(b)** K91A/K325G. Symbols denote (C) crude enzyme, flow-through of impurities and washing step fractions. The elution fractions represented GST-tagged mutant lipases (~69 KDa). GST tag was cleaved by PreScission Protease. Purified protein denotes purified mutant lipases (~43 KDa). GST tag was collected into later fractions (~26 kDa). A standard protein marker (Fisher Thermo Scientific, USA) was utilised.

Table 1. Predicted structure validation of WT and mutant lipases based on Ramachandran plot and Errat2.

	The structure quality						
**Validation tools**	WT	Single mutants			Double mutants		
		K91A	K177A	K325G	K91A/ K177A	K91A/ K325G	K177A/ K325G
**Ramachandran Plot**							
**a) Most favored region**	90.6%	90.9%	92.7%	92.1%	87.2%	92.1%	92.1%
**b) Additional allowed region**	9.1%	8.8%	6.4%	7.0%	11.9%	7.3%	7.3%
**c) Generously allowed region**	0.3%	0.3%	0.9%	0.9%	0.9%	0.3%	0.6%
**d) Disallowed region**	0.0%	0.0%	0.0%	0.0%	0.0%	0.3%	0.0%
**Errat2 (Overall quality factor)**	97.60	98.39	99.73	98.93	92.61	95.21	96.78

Table S2. QMEAN analysis of WT and the mutants. The analysis of QMEAN Z-scores consist of linear combination from six structural descriptors as scoring function to show the overall structure quality for each of the enzymes. Another four Z-scores of individual analysis were reported.

Lipases	QMEAN	C−β	All Atom	Solvation	Torsion
WT	−0.64	−0.48	−1.04	−0.41	−0.56
K91A	−0.46	−0.46	−1.27	−0.49	−0.19
K177A	−0.84	−0.87	−0.91	−0.38	−0.61
K325G	−0.83	−0.72	−0.90	−0.21	−0.65
K91A/K177A	−1.68	−0.85	−1.30	−0.94	−1.27
K91A/K325G	−0.66	−1.02	−1.08	−0.70	−0.33
K177A/K325G	−0.64	−0.82	−1.22	−0.65	−0.34
